# Fetal and infant exposure to severe Chinese famine increases the risk of adult dyslipidemia: Results from the China health and retirement longitudinal study

**DOI:** 10.1186/s12889-017-4421-6

**Published:** 2017-06-14

**Authors:** Zhenghe Wang, Changwei Li, Zhongping Yang, Jun Ma, Zhiyong Zou

**Affiliations:** 1 Institute of Child and Adolescent Health, School of Public Health, Peking University Health Science Center, No 38 Xue Yuan Road, Haidian District, Beijing, 100191 China; 2 Department of Epidemiology, Tulane University School of Public Health and Tropical Medicine, New Orleans, LA USA

**Keywords:** Chinese famine, Fetal malnutrition, Dyslipidemia, Gender difference

## Abstract

**Background:**

To explore the associations between the Chinese famine exposure in early life and the dyslipidemia in adulthood.

**Methods:**

We selected 2752 participants from the baseline survey of China Health and Retirement Longitudinal Study (CHARLS) 2011–2012 to evaluate the associations of early life the Chinese famine exposure with risk of dyslipidemia in adulthood. Dyslipidemia was defined as TC (Total Cholesterol): HDL-C (High-Density Lipoprotein Cholesterol) ratio ≥ 5.0 or use cholesterol lowering drugs. Famine exposure cohorts were categorized by birthdates of participants. Binary logistics regression model was used to examine the associations of early-life famine exposure with the risk of dyslipidemia.

**Results:**

The dyslipidemia prevalence of the non-exposed cohort, fetal stage-, infant stage-, and preschool stage-exposed cohorts in adulthood was 15.7%, 23.1%, 22.0%, and 18.6%, respectively. Early-life exposure to the Chinese famine significantly increased LDL cholesterol concentrations in adulthood after adjusted for age. The risks of dyslipidemia in fetal (OR = 1.58; 95% CI: 1.23–2.03; *P* < 0.001) and infant (OR = 1.52; 95% CI: 1.15–2.00; *P* = 0.003) stage-exposed cohorts were significantly higher than the non-exposed cohort after adjusted for gender and current family economic status. Following gender stratification, we found that fetal (OR = 1.80; 95% CI: 1.26–2.57; *P* = 0.001), infant (OR = 1.75; 95% CI: 1.17–2.62; *P* = 0.006), and preschool (OR = 1.63; 95% CI: 1.10–2.42; *P* = 0.015) -stage exposure to severe famine aggravated the risk of dyslipidemia in female adults. However, the similar association was not observed for male adults.

**Conclusions:**

Early-life exposure to severe Chinese famine could link with the higher dyslipidemia risk in female adulthood, but not in male adulthood. This gender-specific effect might be associated with the hypothesis that parents in China prefer boys to girls traditionally or survivors’ bias.

**Electronic supplementary material:**

The online version of this article (doi:10.1186/s12889-017-4421-6) contains supplementary material, which is available to authorized users.

## Background

Dyslipidemia is an important risk factor of coronary heart disease (CHD), which is one of the leading causes of death in developing and developed counties [1]. Based on the Chinese national data, the prevalence of dyslipidemia was 26.7% among workers aged 18–59 years in 2012 [2], and was 33.5% among Chinese aged greater than 45-years-old [3], which was higher than those among American population (29.3%) aged 45 to 84 years [4]. The emerging pandemic was partially caused by population growth, rapidly aging and changes of diet and lifestyle [2, 3, 5]. However, recent studies indicated that the early life famine exposure also might increase the later susceptibility to some common chronic diseases [6], including metabolic syndrome [7], diabetes [8], and fatty liver disease [9].

The early origins of disease hypothesize that adaptability change for early-life (fetal, infant and early-child stage) severe malnutrition could result in bodily changes. Although these adaptability changes could contribute to early-life survival, they may elevate the risk of some common metabolic diseases in later life [10, 11]. Many animal model studies had supported the hypothesis in the past decades, but direct human evidence is rare due to ethical limitations. However, historical famine provided us a unique environment to examine the effect of early-life severe famine exposure on adverse health outcomes in adulthood [12]. Over the last few decades, many studies focused on the Dutch famine, and found that the infant stage exposure to famine increased the risks of hypertension [13], cardiovascular disease [14], diabetes [15], and other diseases [16] in adulthood. However, the studies focused on dyslipidemia were extremely limited and results were contradictory. According to our knowledge, just two studies based on European explored the association between early-life famine exposure and dyslipidemia [17, 18]. One Dutch famine study reported that only female prenatal malnutrition was associated with the elevated total cholesterol and triglycerides concentrations, but not with dyslipidemia [17]. However, the other study from Israel found positive association between early-life famine exposure and dyslipidemia [18].

Because of the radical collectivization movement and inclement climate conditions, almost the entire Mainland China suffered from extreme food shortage during 1959–1961 [19, 20]. Different from the Dutch famine, the Chinese famine was more severe, lasted for a longer period and far-reaching, leading to about 30 million premature deaths [21]. In addition, food supplies recovered slowly after the Chinese famine due to low social economic condition [22]. Apart from the result in large-scale premature deaths, the Chinese famine also exhibited a severe adverse effect on the later health of the survivors. For example, several studies have found that in severe famine affected areas, individuals who exposure to the Chinese famine in early life significantly increased the risks of adult diabetes [8], metabolic syndrome [7], which did not happen in less severe affected areas. Our recent study had demonstrated that the infant stage exposure to the severe Chinese famine substantially elevated the risk of hypertension in later life [23]. However, the association between Chinese famine exposure in early life and the dyslipidemia in adulthood has not been reported yet.

In the current study, the China Health and Retirement Longitudinal Study (CHARLS) 2011–2012 baseline databases were used to explore whether different stages and severity of famine exposure in early-life were associated with the dyslipidemia in adulthood, and to examine the gender difference.

## Methods

### Participants

**Fig. 1:**
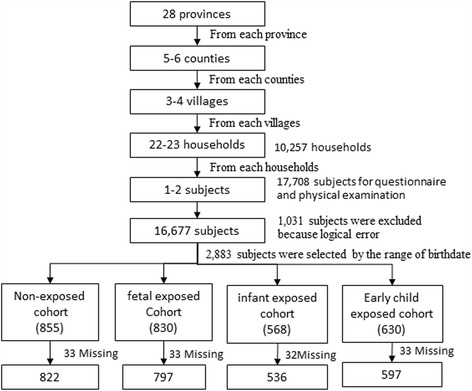
Flowchart on the sample selecting methods at each step

The CHARLS is a longitudinal large-scale national survey of the middle-aged and elderly Chinese population and was followed up every two years. The baseline data collection of the survey was performed from June 2011 to March 2012. The protocol was the same as before [24]. Briefly, 17,708 participants aged more than 45 years were selected from 10,257 households through four-staged, stratified, cluster sampling among 28 provinces, municipalities, and autonomous regions in Mainland China. In the current study, 2883 participants were enrolled into four cohorts as defined by birthdates. After excluding 131 participants with missing values in blood lipid profile, 2752 subjects participated in the current study (Fig. 1).

### Defining famines cohorts

We categorized all the subjects by birthdate into four birth cohorts based on the life stage when the famine happened: non-exposed cohort, fetal stage-exposed, infant stage-exposed, and preschool stage-exposed cohorts. Because the Chinese famine lasted for three years from 1959 to 1961, but the exact dates of famine starting and ending were not clear. In order to minimize the misclassification bias, participants born between January 1st, 1959 and September 30th, 1959, and between October 1st, 1961 and September 30th, 1962 were removed. The participants’ birthdate ranges of four cohorts were the same with our previous paper [23] and were listed briefly below: the non-exposed cohort (10/01/1962–09/30/1964), the fetal stage-exposed cohort (10/01/1959–09/30/1961), the infant stage-exposed cohort (01/01/1958–12/31/1958), the preschool stage-exposed cohort (01/01/1956–12/31/1957). The participant’s mean age (standard deviation) of the four cohorts was 47.4 (0.5), 50.9 (0.7), 53.2 (0.4), and 55.1 (0.4) years, respectively.

### Defining famine areas

As described [23], although the Chinese famine affected all the provinces in China, the famine severity was different in different provinces due to variation of climate, population density, and policies regarding shortage of foods [25]. Referring to the method in previous studies [23, 25], the current study also used excess mortality to partly reflect the severity of the famine exposure. The computing method of excess death rate was described in our previous study [23]. An excess mortality of 50% was used to differentiate the severe affected areas from less affected areas in the present study.

### Diagnosis of dyslipidemia

Frozen plasma total cholesterol (TC), high-density lipoprotein cholesterol (HDL-c), low-density lipoprotein cholesterol (LDL-c), and triglycerides (TG) from venous blood were examined by the standard enzymatic colorimetric method [26–28]. The classifications from the Guidelines for The China Adult Dyslipidemia Prevention, Evaluation and Treatment [29] were used to define elevated TC (TC ≥6.216 mmol/L; 240 mg/dL), low HDL-c (HDL <1.036 mmol/L; 40 mg/dL), elevated LDL-c (LDL-c ≥ 4.138 mmol/L; 160 mg/dL), and elevated TG (TG <2.250 mmol/L; 200 mg/dL). We defined dyslipidemia as TC/HDL-c > 5.0 [30] or self-report dyslipidemia to avoid the influence of lipid-lowering drugs. The participants were recognized as suffering from self-reported dyslipidemia if they answered ‘yes’ to the question below: Have you been diagnosed with the dyslipidemia (elevated LDL-c, TG, and TC, or a low HDL-c) by a registered physician?

### Covariates

The mean family income (12,000 Chinese Yuan per person per year) of the current samples was selected as a threshold to divide all participants into two groups (low economic status group and high economic status group) [31]. The body mass index (BMI) was calculated using weight in kilometer divided by square of height in meter, and 24.0 kg/m^2^ of BMI was used as a threshold (recommended for Chinese adults) to divide all participants into normal weight and overweight/obese groups. Actual age of the participants was calculated when detect time subtracted the birthdates.

### Statistical analysis

Data were collected by computer-aided personal survey (CAPI) system. SPSS version 20.0 (SPSS Inc. Chicago, IL) was used for the statistical analysis.

ANOVA and ANCOVA with TC, TG, HDL, and LDL, respectively as dependent variables and age as a covariant were conducted to test the difference of TC, TG, HDL, and LDL among the four birth cohorts. The Dunnett-t test was used to perform multiple comparisons between the three exposed cohorts and the non-exposed cohort, respectively. Logarithmic transformation was used to transfer the asymmetrical distribution data to a normal distribution or near-normal distribution.

The method of maximum likelihood by the binary logistics regression model, control for gender, and current family economic status, was used to examine the risks of dyslipidemia among fetal, infant, and preschool stage-exposed cohorts, where the non-exposed cohort was the reference group. The interaction between the famine exposure cohorts and famine effect areas, BMI, and gender was examined by providing a multiplicative factor in the logistics regression model. The analyses were controlled for sex and the current family economic status.

To explore whether the associations of early-life the Chinese famine exposure with the dyslipidemia were sex-specific, the stratified analysis by sex was performed in adulthood.

## Results

The social demographic characteristics of participants were presented in Table 1. A total of 2752 subjects were enrolled into the current study, 797 participants had been exposed to the Chinese famine during fetal stage, whereas 536 and 597 participants had been exposed to the famine during infant and preschool stage, respectively. The distribution of gender and areas did not demonstrate any statistical difference among the four birth cohorts. On the other hand, the difference was observed in the mean concentration of plasma TC, TG, and LDL cholesterol. However, consistency in the HDL cholesterol was not seen. After adjusted for age, we found that only the LDL-c level of fetal (*d* = 0.40, 95% CI: 0.09–0.71; *P* = 0.011), infant (*d* = 0.61, 95% CI: 0.12–1.09; *P* = 0.014), and preschool (*d* = 0.78, 95% CI: 0.14–1.42; *P* = 0.017) stage-exposed cohorts were obviously higher than the non-exposed cohort.

**Table 1:** Basic characteristics of the study population according to the Chinese famine exposure

Variables	Non-exposed cohort 10/1/1962–9/30/1964 (*N* = 822)	Fetal-exposed cohort 10/1/1959–9/30/1961 (*N* = 797)	Infant-exposed cohort 1/1/1958–12/30/1958 (*N* = 536)	Preschool-exposed cohort 1/1/1956–12/30/1957 (*N* = 597)
Gender *n* (%)				
Male	383 (46.5)	381 (47.9)	277 (51.7)	310 (52.0)
Female	439 (53.5)	415 (52.1)	259 (48.3)	286 (48.0)
Area *n* (%)				
Severely	514 (62.5)	495 (62.1)	339 (63.2)	377 (63.1)
Less severely	308 (37.5)	302 (37.9)	197 (36.8)	220 (36.9)
Smoking n (%)^**^				
Never smoking	528 (64.6)	487 (62.0)	310 (58.2)	320 (53.7)
Former smoking	79 (9.7)	72 (9.2)	59 (11.1)	77 (12.9)
Current smoking	210 (25.7)	227 (28.9)	164 (30.8)	199 (33.4)
Drinking n(%)				
Never drinking	578 (70.9)	548 (69.7)	377 (70.9)	397 (66.6)
Former drinking	8 (1.0)	8 (1.0)	10 (1.9)	14 (2.3)
Current drinking	229 (28.1)	230 (29.3)	145 (27.3)	185 (31.0)
BMI mean(SD) kg/m2^**^	24.40 (3.90)	24.29 (4.15)	23.73 (3.81)^†^	23.48 (3.58)^‡^
Age mean(SD) year^**^	47.37 (0.48)	50.91 (0.67)^‡^	53.17 (0.37)^‡^	55.14 (0.35)^‡^
TC mean(SD) mmol/L^**^	4.77 (0.91)	4.98 (0.95)^‡^	4.99 (0.94)^‡^	5.01 (0.99)^‡^
TG mean(SD) mmol/L^*^	1.30 (1.79)	1.39 (1.83)	1.28 (1.75)	1.25 (1.71)
HDL mean(SD)mmol/L	1.23 (1.31)	1.20 (1.37)	1.24 (1.39)	1.27 (1.34)
LDL mean(SD)mmol/L^**,#^	2.79 (0.82)	2.95 (0.93)^†, $^	2.99 (0.86)^‡, $^	3.02 (0.86)^‡, $^

*TC* total cholesterol, *TG* triglyceride, *HDL* high density lipoprotein cholesterol, *LDL* low density lipoprotein cholesterol, *BMI* body mass index, *SD* standard deviation

^*^Mean values or distribution were significantly different among the four birth cohorts (ANOVA or χ^2^-test; *P* < 0.01)

^**^Mean values or distribution were significantly different among the four birth cohorts (ANOVA or χ^2^ test; *P* < 0.001)

^†^Mean values were significantly different between exposed cohort and non-exposed cohort (Dunnett’s, t-test, *P* < 0.05)

^‡^Mean values were significantly different between exposed cohorts and non-exposed cohort (Dunnett’s, t-test, *P* < 0.01)

^#^Mean values were significantly different as assessed by ANCOVA with lipid profile as a dependent variable and age as a covariate among the four birth cohorts (*P* < 0.05)

^$^Mean values were significantly different by ANCOVA with lipid profiles as a dependent variable and age as a covariate between exposed cohorts and non-exposed cohort (*P* < 0.05)

Table 2 presented the risk of dyslipidemia, elevated TC, elevated LDL cholesterol, low HDL cholesterol, and elevated TG of famine exposed cohorts compared with the non-exposed cohort, using the binary logistic regression model. The dyslipidemia prevalence of the non-exposed cohort, fetal-, infant-, and preschool-exposed cohorts was 15.7%, 23.1%, 22.0%, and 18.6%, respectively. Compared with the non-exposed cohort, the fetal (OR 1.58; 95% CI: 1.23–2.03; *P* < 0.001), and infant (OR 1.52; 95% CI: 1.15–2.00; *P* = 0.003) stage-exposed cohorts revealed a significantly higher risk of dyslipidemia after adjusted for gender and current family economic status. The same methods were conducted to analyze the risk of elevated TC, elevated LDL cholesterol, low HDL cholesterol, and elevated TG, respectively. We observed that all the famine-exposed cohorts significantly increased the risks of elevated TC (≥ 240 mg/dl) and elevated LDL cholesterol (≥ 260 mg/dL) after adjusted for gender and current family economic status compared with the non-exposed cohort. However, a consistent association for elevated TG (≥ 200 mg/dL) and low HDL cholesterol (< 40 mg/dL) was not noted.

**Table 2:** Risk of dyslipidemia among famine exposed cohorts compared with the non-exposed cohort

Variables	Non-exposed cohort	Fetal-exposed cohort	Infant-exposed cohort	Preschool-exposed cohort
Elevated TC ≥ 6.216 mmol/L; 240 mg/dL (%)	4.3	7.5	6.9	7.5
*P* ^a^		0.006	0.035	0.009
Odds ratio (95% CI)^a^	Ref.	1.83 (1.19–2.81)	1.67 (1.04–2.68)	1.83 (1.16–2.89)
*P* ^b^		0.007	0.028	0.006
Odds ratio (95% CI)^b^	Ref.	1.80 (1.17–2.77)	1.71 (1.06–2.75)	1.89 (1.23–2.31)
Low HDL cholesterol <1.036 mmol/L; 40 mg/dL (%)	15.9	18.9	16.6	15.7
*P* ^a^		0.111	0.744	0.922
Odds ratio (95% CI)^a^	Ref.	1.23 (0.95–1.60)	1.05 (0.78–1.41)	0.92 (0.74–1.32)
*P* ^b^		0.152	0.867	0.816
Odds ratio (95% CI)^b^	Ref.	1.21 (0.93–1.56)	1.03 (0.76–1.38)	0.97 (0.72–1.29)
Elevated TG ≥ 2.25 mmol/L; 200 mg/dL (%)	10.1	12.8	10.1	10.1
*P* ^a^		0.088	0.989	0.977
Odds ratio (95% CI)^a^	Ref.	1.31 (0.96–1.78)	1.00 (0.70–1.43)	1.00 (0.70–1.41)
*P* ^b^		0.136	0.919	0.924
Odds ratio (95% CI)^b^	Ref.	1.27 (0.93–1.72)	0.92 (0.68–1.41)	0.98 (0.69–1.40)
Elevated LDL cholesterol ≥4.138 mmol/L; 260 mg/dL (%)	3.4	7.3	6.0	6.4
*P* ^a^		0.001	0.026	0.010
Odds ratio (95% CI)^a^	Ref.	2.23 (1.40–3.53)	1.80 (1.07–3.03)	1.93 (1.17–3.18)
*P* ^b^		0.001	0.022	0.008
Odds ratio (95% CI)^b^	Ref.	2.20 (1.38–3.49)	1.84 (1.09–3.10)	1.98 (1.20–3.27)
Prevalence dyslipidemia: TC*/*HDL cholesterol Ratio > 5 or self-report dyslipidemia (%)	15.7	23.1	22.0	18.6
*P* ^a^		<0.001	0.003	0.151
Odds ratio (95% CI)^a^	Ref.	1.62 (1.27–2.09)	1.53 (1.16–2.02)	1.23 (0.93–1.62)
*P* ^b^		<0.001	0.003	0.169
Odds ratio (95% CI)^b^	Ref.	1.58 (1.23–2.03)	1.52 (1.15–2.00)	1.22 (0.92–1.61)

*CI* Confidence Interval, *Ref* Reference

^a^Evaluating the overall risk of three famine exposed cohorts with non-exposed as a reference by the binary logistic regression model

^b^Evaluating the risk of three exposed cohorts with non-exposed as a reference by the binary logistic regression model after adjusted for gender and current family economic status

Table 3 presented the dyslipidemia prevalence and risk of the exposed cohorts compared with the non-exposed cohort stratified by severity of the famine across the entire Mainland China. In severely affected areas, the dyslipidemia prevalence among non-exposed cohort, fetal-period, infant-period, and preschool-exposed cohorts were 15.6%, 23.6%, 21.8%, and 19.6%, respectively. In less severely affected areas, the prevalence of dyslipidemia among the four birth cohorts was 15.9%, 22.5%, 22.8%, and 16.8%, respectively. Compared with the non-exposed cohort, a significantly higher dyslipidemia risk for fetal (OR = 1.63; 95% CI: 1.19–2.24; *P* = 0.002) and infant (OR = 1.50; 95% CI: 1.05–2.13; *P* = 0.024) stage-exposed cohorts was observed after adjusted for gender and current family economic status in severely affected areas. However, consistent results were not observed in the preschool stage-exposed cohort or less severely affected areas. In addition, significant interaction was not observed between exposed cohort and areas for the all the famine-exposed cohorts, even after adjusted for gender and current family economic status. However, a significant interaction was observed between exposed cohort and BMI for all the famine-exposed cohorts (Additional file 1: Table S1).

**Table 3:** Prevalence of dyslipidemia in birth cohorts of the Chinese famine areas

Variables	Non-exposed cohort	Fetal-exposed cohort	Infant-exposed cohort	Preschool-exposed cohort
Severely affected famine area				
Prevalence (%)	15.6	23.6	21.8	19.6
*P* ^a^		0.001	0.020	0.114
Odds ratio (95% CI)^a^	Ref.	1.68 (1.22–2.30)	1.52 (1.07–2.15)	1.33 (0.94–1.88)
*P* ^b^		0.002	0.024	0.124
Odds ratio (95% CI)^b^	Ref.	1.63 (1.19–2.24)	1.50 (1.05–2.13)	1.32 (0.93–1.87)
Less severely affected famine area				
Prevalence (%)	15.9	22.5	22.8	16.8
*P* ^a^		0.039	0.050	0.780
Odds ratio (95% CI)^a^	Ref.	1.54 (1.02–2.31)	1.57 (1.00–2.46)	1.07 (0.67–1.71)
*P* ^b^		0.058	0.063	0.827
Odds ratio (95% CI)^b^	Ref.	1.49 (0.84–2.18)	1.41 (0.82–2.40)	1.07 (0.61–1.86)
*P* for interaction between area and cohort^a^	Ref.	0.995	0.640	0.168
*P* for interaction between area and cohort^b^	Ref.	0.950	0.583	0.122

*CI* Confidence Interval, *Ref* Reference

^a^Evaluating the overall risk of three famine exposure cohorts with non-exposed as a reference by the binary logistic regression model

^b^Evaluating the risk of three famine exposure cohorts with non-exposed as reference by the binary logistic regression model after adjusted for gender and current family economic status

Stratified analysis by gender was showed in Table 4. For male participants, the dyslipidemia prevalence of non-exposed, fetal, infant, and preschool exposed cohorts was 17.8%, 23.4%, 22.4%, and 16.5%, respectively. We did not observe a significant difference between non-exposed cohort and three famine-exposed cohorts. For female participants, the dyslipidemia prevalence of non-exposed, fetal, infant, and preschool exposed cohorts was 13.9%, 22.9%, 22.0%, and 21.0%, respectively. Compared with the non-exposed cohort, fetal (OR = 1.80; 95% CI: 1.26–2.57; *P* = 0.001), infant (OR = 1.75; 95% CI: 1.17–2.62; *P* = 0.006), and preschool (OR = 1.63; 95% CI: 1.10–2.42; *P* = 0.038) stage-exposed cohorts significantly increased the risk of dyslipidemia. Furthermore, a significant interaction was observed between gender and preschool-exposed cohort (*P* = 0.038).

**Table 4:** Prevalence rate of dyslipidemia by gender and birth cohorts

Variables	Non-exposed cohort	Fetal-exposed cohort	Infant-exposed cohort	Preschool-exposed cohort
Male				
Prevalence (%)	17.8	23.4	22.4	16.5
*P* ^a^		0.058	0.145	0.64
Odds ratio (95%CI)^a^	Ref.	1.41 (0.99–2.00)	1.33 (0.91–1.96)	0.91 (0.61–1.35)
*P* ^b^		0.080	0.174	0.617
Odds ratio (95%CI)^b^	Ref.	1.37 (0.96–1.96)	1.31 (0.89–1.93)	0.90 (0.61–1.35)
Female				
Prevalence (%)	13.9	22.9	22.0	21.0
*P* ^a^		0.001	0.006	0.013
Odds ratio (95%CI)^a^	Ref.	1.84 (1.29–2.62)	1.75 (1.17–2.61)	1.65 (1.11–2.44)
*P* ^b^		0.001	0.006	0.015
Odds ratio (95%CI)^b^	Ref.	1.80 (1.26–2.57)	1.75 (1.17–2.62)	1.63 (1.10–2.42)
*P* for interaction between gender and cohort^a^		0.294	0.336	0.038
*P* for interaction between gender and cohort^b^	Ref.	0.281	0.301	0.038

*CI* Confidence Interval, *Ref* Reference

^a^Evaluating the overall risk of three exposed cohorts with non-exposed as a reference by the binary logistic regression model

^b^Evaluating the risk of three exposed cohorts with non-exposed as a reference by the binary logistic regression model after adjusted for current family economic status

## Discussion

In the present study, we observed that fetal and infant stage exposure to severe Chinese famine significantly increased the risk of dyslipidemia in adulthood. After stratified by gender, it was observed that fetal, infant, and preschool stage famine exposure could elevate the risk of dyslipidemia in female adults, but not in male adults. These results suggested that early life might be a critical period in human lipid metabolism and development.

Several mechanisms might explain the link of famine exposure in early life with the elevated dyslipidemia risk in later life. 1) Severe maternal under-nutrition status during pregnancy could alter the synthesis of cholesterol and increase the plasma concentration of cholesterol [32] which has been proved by animal experiments. 2) Individuals who suffered from severe intrauterine malnutrition are more likely to consume a high-fat diet in adulthood while reducing the level of physical activity, thereby increasing atherogenic lipid profile, as observed by a Dutch famine study [33]. In the present study, we did not explore the correlation between famine exposure and dietary and physical behavior due to lack of relevant data. 3) Lipid metabolic gene methylation level may play a pivotal role in malnutrition during pregnancy, which renders its sensitive to abnormal lipid metabolism [34]. Elmar et al. comprehensively assessed the associations of prenatal malnutrition and differentially methylated regions (P-DMRs) in humans and found that *CPTIA* was involved in fatty acid oxidation and *KLF13* involved in cholesterol metabolism [35]. The methylation level as one of principal components of the epigenetic, could effectively adjust the expression level of the gene [36]. In addition, epigenetic plasticity may play an important role in postnatal period and result in the “metabolic imprinting” that could be another biological mechanism beneath the association of early life malnutrition with metabolic diseases in adulthood [37, 38].

In the present study, we observed that dyslipidemia prevalence for those suffering from severe malnutrition in the fetal and infant stage were significantly higher than the non-exposed group, which was similar with a previous study [18]. However, the dyslipidemia prevalence (80.7% in fetal or early infant stage exposed group and 72.4% in non-exposed group) [18] was significantly higher than our results (23.1% in fetal stage exposed cohort and 15.7% in the non-exposed cohort). This difference may be attributable to several reasons. Firstly, age and race of subjects are different between the two studies. The subjects of the current study were Asian population and the other study aimed at European population, and the mean age was significantly younger in the current study (52 years) than the other study (mean age was 69 years) [7]. Secondly, the diagnosis standard was different between the two studies. In the current study, the dyslipidemia prevalence was based on self-reported data and TC/HDL cholesterol >5.0, while the European study based on physician diagnoses and medical prescription database of the Community Health Service (CHS).

The present study focused on dyslipidemia as the primary outcome and found that fetal and infant stage exposure to the severe Chinese famine significantly increased the risk of dyslipidemia in later life. Until now, no study directly assessed the effect of the Chinese famine exposure in early life on dyslipidemia in adulthood. However, Li and his colleagues focused on the metabolic syndrome demonstrated that the risk of metabolic syndrome in fetal stage exposure to severe Chinese famine was 2.13-fold higher than the non-exposed cohort in later life [39]. These associations showed that fetal and infant stages could be critical periods for the propensity to dyslipidemia in adulthood.

In the current study, we observed that fetal, infant, and preschool stage exposure to the Chinese famine significantly increased the level of LDL cholesterol, but no consistent result was observed for HDL cholesterol, total cholesterol, and triglyceride. A Dutch famine study showed that male exposure to famine in early gestation significantly decreased the HDL-cholesterol level (0.08 mmol/L; 0.00, 0.14 mmol/L) than those unexposed [40], which was in disagreement with our results. Thus, we speculated that it might be linked with race, severity, and fat shortage in food between the Dutch and Chinese famines.

After stratified analysis by gender, we found that early-life stage exposure to the Chinese famine significantly elevated the risk of dyslipidemia in female adults, but not in male. It was consistent with the results from study of Wang [41], which observed that only female fetal- and childhood-exposed cohorts significantly increased the risk of metabolic syndrome in later life (*P* < 0.05).

We speculated that the culture differences between European and Chinese populations could be a main reason for this gender difference. In China, male children took precedence over the female due to gender bias, and thus they may potentially be sufficiently nourished during the famine [42]. Additionally, survivor bias might be another reason to interpret the lack of “effect” on males. Males were more vulnerable prenatal insult than females to both short and long term effects of famine [43, 44], but die at higher rates [45]. Thus, male survivors could be healthier than female survivors [46].

### Strengths and limitations

This study has several limitations. Firstly, the selection bias caused by excess mortality in early life could be a main limitation. Subjects with abnormal metabolism and structure may succumb to the famine, leaving only the stronger and healthier participants. The bias may lower the real effect of famine exposure on dyslipidemia, and thus it could not overestimate the link of the famine exposure with dyslipidemia. Secondly, we cannot completely divide the fetal exposed cohort from infant exposed cohort because the Chinese famine lasted for three years (1959–1961). A lack of an adequate method accurately distinguishes whether they were fetal or infant exposed to the Chinese famine. However, in this study, subjects who born from January 1, 1958 to December 31, 1958 was defined as the infant-exposed cohort to ensure that the vast majority of the subjects in this cohort were exposed to Chinese famine in an infant stage. Furthermore, we also found that both the fetal and infant stage famine exposure increased the risk of dyslipidemia in later life. Thirdly, the excess death rate was used to evaluate the severity of suffering from the Chinese famine in this study. With this indicator, the confounding factors, such as adverse climate conditions or infections that may also contribute to the deaths are not taken into consideration. Also, the information of personal energy intake during famine period was not available in the current study. Thus, we cannot accurately attribute the effect of famine exposure in early life on elevated risks of dyslipidemia. Additional, similar with the other studies focused on the Chinese famine [25], the current study also lacked the objective indexes, such as birth weight and length to reflect the effect of famine exposure on physical health outcomes. Although the current study was open to residual confounding or selection bias, it used a national data with broad representativeness, combining self-report dyslipidemia with serum lipid profile examination to evaluate the association between early-life the Chinese famine exposure and dyslipidemia in later life and found that early life exposed to severe malnutrition could be associated with the higher risk of adult non-communicable diseases. Despite China has been food secure now; many people are still exposed to food shortages in low-income and middle-income countries [47, 48]. Hence many children are still at short term risk of malnutrition associated mortality and morbidity [47], but also at long term risk of adult non-communicable diseases. The heavily short and long term adverse effects from severe food shortage ask us to implement the urgent interventions to lower their occurrence or improve their consequences.

## Conclusions

The present study found that female fetal and infant stages exposure to the severe Chinese famine had an elevated risk of dyslipidemia in adulthood.

## Additional file

Additional file 1: Table S1. Stratified analysis by BMI for dyslipidemia prevalence in birth cohorts of the Chinese famine areas. **Table S1.** showed the results of stratified analysis by BMI for dyslipidemia prevalence in birth cohorts of the Chinese famine areas. (DOCX 17 kb)

## Abbreviations

BMI: Body mass index
CHARLS: Chinese health and retirement longitudinal study
CHD: Coronary heart disease
CI: Confidence interval
HDL: High-density lipoprotein
LDL: Low-density lipoprotein cholesterol
OR: Odds ratio
TC: Total cholesterol
TG: Triglyceride
