# Supplementary material for: Fetal and infant exposure to severe Chinese famine increases the risk of adult dyslipidemia: Results from the China health and retirement longitudinal study
Source: BMC Public Health. 2017 Jun 14;17:488. doi: 10.1186/s12889-017-4421-6 (PMC5470181; doi:10.1186/s12889-017-4421-6)
Supplement: Additional file 1: Table S1. — Stratified analysis by BMI for dyslipidemia prevalence in birth cohorts of the Chinese famine areas. Table S1. showed the results of stratified analysis by BMI for dyslipidemia prevalence in birth cohorts of the Chinese famine areas. (DOCX 17 kb) [file 12889_2017_4421_MOESM1_ESM.docx]

**Additional file 1: Table S1.** Stratified analysis by BMI for dyslipidemia prevalence in birth cohorts of the Chinese famine areas

| Variables | Non-exposed cohort | Fetal-exposed cohort | Infant-exposed cohort | Preschool-exposed cohort |
| --- | --- | --- | --- | --- |
| BMI≥24.0 kg/m^2^ |  |  |  |  |
| Prevalence (%) | 8.5 | 15.8 | 13.7 | 11.4 |
| *P^a^* |  | 0.014 | 0.039 | 0.058 |
| Odds ratio (95%CI) *^a^* | Ref. | 1.59(1.10-2.29) | 1.54(1.02-2.33) | 1.50(0.99-2.86) |
| *P^b^* |  | 0.012 | 0.039 | 0.062 |
| Odds ratio (95%CI)*^b^* | Ref. | 1.61(1.11-2.32) | 1.55(1.02-2.34) | 1.50(0.98-2.89) |
| BMI<24.0 kg/m^2^ |  |  |  |  |
| Prevalence (%) | 24.5 | 33.9 | 33.3 | 32.7 |
| *P^a^* |  | 0.007 | 0.059 | 0.263 |
| Odds ratio (95%CI)*^a^* | Ref. | 2.01（1.21-3.34） | 1.71(0.98-2.97) | 1.37(0.79-2.39) |
| *P^b^* |  | 0.007 | 0.05 | 0.245 |
| Odds ratio (95%CI)*^b^* | Ref. | 2.03(1.22-3.38) | 1.74(1.00-3.04) | 1.39(0.80-2.43) |
| *P* for interaction between BMI and cohort *^a^* | Ref. | <0.001 | <0.001 | <0.001 |
| *P* for interaction between BMI and cohort *^b^* | Ref. | <0.001 | <0.001 | <0.001 |

CI, Confidence Interval; Ref, Reference

*^a^* Evaluating the overall risk of three famine exposure cohorts with non-exposed as a reference by the binary logistic regression model.

*^b^* Evaluating the risk of three famine exposure cohorts with non-exposed as reference by the binary logistic regression model after adjusted for gender, current family economic status, and areas.
